# Similar levels of disease activity and remission rates in patients with psoriatic arthritis and rheumatoid arthritis—results from the Finnish quality register

**DOI:** 10.1007/s10067-023-06850-y

**Published:** 2023-12-28

**Authors:** Lauri Weman, Henri Salo, Laura Kuusalo, Johanna Huhtakangas, Johanna Kärki, Paula Vähäsalo, Maria Backström, Tuulikki Sokka-Isler

**Affiliations:** 1 University of Eastern Finland, Kuopio, Finland; 2 Data and Analytics, Finnish Institute for Health and Welfare (THL), Helsinki, Finland; 3 Department of Internal Medicine, University of Turku and Turku University Hospital, Turku, Finland; 4 Division of Rheumatology, Kuopio University Hospital, Kuopio, Finland; 5 Department of Children and Adolescents, Kanta-Häme Central Hospital, Hämeenlinna, Finland; 6 Research Unit of Clinical Medicine, University of Oulu, Department of Children and Adolescents, Oulu University Hospital and Medical Research Center, Oulu University Hospital and University of Oulu, Oulu, Finland; 7 Department of Paediatrics, Wellbeing Services County of Ostrobothnia, Vaasa, Finland; 8 Research Unit of Clinical Medicine, University of Oulu, Oulu, Finland; 9 Rheumatology, Hospital Nova of Central Finland, Jyväskylä, Finland

**Keywords:** Arthritis, Disease activity, Remission rates

## Abstract

**Objectives:**

To compare the current disease activity and remission rates, and their regional variation in patients with psoriatic arthritis (PsA) and rheumatoid arthritis (RA) in Finland.

**Methods:**

Data of patients’ most recent visit in 1/2020–9/2021 were extracted from the Finnish Rheumatology Quality Register. Measures for disease activity and remission included joint counts, DAS28, cDAPSA, CDAI, the Boolean definition, and physician assessment. Regression analyses were applied, adjusted for age and sex.

**Results:**

Data of 3598 patients with PsA (51% female, mean age 54 years) and 13,913 patients with RA (72% female, 74% ACPA-positive, mean age 62 years) were included. The median (IQR) DAS28 was 1.9 (1.4, 2.6) in PsA and 2.0 (1.6, 2.7) in RA (*p* = 0.94); for cDAPSA, the median (IQR) values were 7.7 (3.1, 14) in PsA and 7.7 (3.3, 14) in RA (*p* < 0.001). In all regions in both diseases, the median DAS28 was ≤ 2.6 and the median cDAPSA < 13. Remission rates included DAS28 < 2.6 in 73% in PsA and 69% in RA (*p* = 0.17) and Boolean remission in 17% in PsA and 15% in RA (*p* < 0.001). By other definitions of remission, the rates ranged between 30% and 46%. Methotrexate was currently used by 49% in PsA and 57% in RA (*p* < 0.001). Self-administered bDMARDs were currently used by 37% in PsA and 21% in RA (*p* < 0.001).

**Conclusion:**

The overall disease activity was low and similar in patients with PsA and RA across the country. Remission rates varied between 15 and 73%, depending on the definition but were similar in PsA and RA.

**Table:** 

**Key Points** *• The disease activity and clinical picture was similar between patients with PsA and RA, in a cross-sectional setting in 1.2020–9.2021.* *• A significant majority of patients with PsA had low disease activity or were in remission according to cDAPSA. Majority of patients with RA were in remission according to DAS28.* *• Patients with PsA and RA used methotrexate similarly. The utilization of bDMARDs was more prevalent in patients with PsA.*

## Introduction

Rheumatoid arthritis (RA) and psoriatic arthritis (PsA) are two common inflammatory rheumatic diseases (IRDs) treated in rheumatology outpatient clinics. When left untreated, the disease course may be progressive, potentially leading to joint damage and impaired functional capacity [1]. Compared to RA, PsA has a more heterogenous clinical picture, affecting the tendons, skin, the axial skeleton, and a larger selection of peripheral joints [2].

Due to the more heterogenous nature of PsA, a lack of validated outcome measures has been a challenge, leaving outcomes research for the disease lagging behind, compared to RA [3]. However, the Disease Activity Score 28 (DAS28) has been used to follow the disease activity of PsA, compromising the joint counts. Disease Activity index for Psoriatic Arthritis (DAPSA), a measurement tool previously used to describe reactive arthritis, was proven to be a sensitive parameter for PsA. Though, C-reactive protein (CRP) is a low value marker to describe the disease activity of PsA and therefore clinical DAPSA (cDAPSA) was developed, to replace its predecessor [4–6]. Nevertheless, these measures are feasible in daily clinical practice, and they provide data on the disease course in individual patients and the status of groups of patients.

Remission has become an achievable goal in RA and PsA in daily clinical practice. However, challenges remain how to define, measure, and report remission. Measures either accept some disease activity [7] or are so stringent that even the general population does not meet the remission criteria [8]. Consensus still has not been reached in defining a clear definition for remission for PsA [1, 7, 9].

Previous studies indicate that disease activity was low in RA and remission rates were among the highest in Finland, in a comparison of 24 countries worldwide [10]. Comparative data on disease activity or remission rates for PsA versus RA do not exist in Finland. Data are sparse worldwide, as uniform validated measures do not exist for both diseases. Nevertheless, we aimed to study the overall current disease status and medication of patients with PsA and RA in Finland, comparing disease activity using DAS28 and cDAPSA across different sex and age groups and regions. We also aimed at comparing remission rates using multiple definitions of remission, which all include general domains such as different joint counts with/without laboratory tests for inflammatory activity, and patient perception.

## Patients and methods

### Database

The source of data. The Finnish Rheumatology Quality Register is kept by the Institute for Health and Welfare (THL). Clinical data are collected using monitoring tools such as GoTreatIT Rheuma (DiaGraphIT, Kristiansand, Norway), BCB (BCB Medical, Turku, Finland), and RaiQu (Vahvero Symbiosis, Kuopio, Finland), which are used in almost all public rheumatology clinics to facilitate treatment decisions and to improve the outcomes of rheumatic diseases. The monitoring tools also serve as a local database for rheumatic diseases, as it contains structural data that can be analyzed for groups of patients for administrative purposes, e.g., THL is mandated to receive the clinical data to the central database at certain intervals, for the purposes of the quality register.

Coverage of the clinical data. Monitoring covers patients who receive care in the rheumatology clinics of the health care regions. In a few rheumatology clinics, monitoring has only started and therefore data from those clinics are limited and not included in the regional comparisons, but they are included in other analyses. Patients who receive care in primary care or private rheumatologists were not included in the database. The 20 health care regions are indicated with a running number, from largest to the smallest, based on the size of the adult population.

Other registers. Other national registers may provide data to the quality register such as the Drug Reimbursement Register and the Drug Purchase Register, which are kept by the Social Insurance Institution of Finland (KELA). Our study used data from the Drug Purchase Register. The databases were merged on individual level, using each individual’s unique identification code.

Coverage of the medication data; limitations. The Drug Purchase Register includes purchases of medications with prescriptions, with date of purchases, amount, and type of each medication. Medication data were extracted since 1st January 2000. However, this register does not include medications that are administered as infusions, because they are purchased by the hospitals and not by the patients directly. Thus, intravenous infliximab and rituximab are not covered by these data. Abatacept and tocilizumab can be administered as infusions and also as subcutaneous injections by patients. Therefore, all treatment courses of these medications are not covered and proportions of patients taking biologic disease-modifying antirheumatic drugs (bDMARDs) cannot be interpreted as absolute percentages within PsA and RA groups. Furthermore, the preference for the route of administration may vary between health care districts. However, within a region, the preferred route of administration of a medication is most probably similar in PsA and RA and therefore comparison of the use of medications between PsA and RA is justified.

### Patients

A total of 3598 adult patients with PsA and 13,913 patients with RA from hospital districts in Finland were identified in the Finnish Rheumatology Quality Register in the daily clinical practice, patients with RA were diagnosed either with the help of 1987 [11] or 2010 ACR/EULAR [12] classification criteria, depending on the year they were diagnosed. Patients with PsA were diagnosed with the help of the psoriatic arthritis (CASPAR) classification criteria [13], when available. All patients were > 16 years old and did not meet the criteria for other types of diseases causing arthritis. The subtype of PsA was not available in the database.

### Patients and index visit

Data from the most recent outpatient visit or remote contact between 1st January 2020 and 30th September 2021 were used; this is the index date for each patient.

### Variables

Demographics. Demographic data included age, sex, disease duration before index visit in years, symptom duration before diagnosis in months, smoking status, smoking history, and work status. Patient work status was obtained primarily from the self-report with response options of working full or part time; not in work force such as student, home maker, maternity/paternity leave; unemployed; work disabled including sick leave, rehabilitation, and permanent work disability.

Laboratory data. A level of < 10 mg/l was considered normal for CRP. Erythrocyte sedimentation rate (ESR) was considered normal as < 20 mm/h for women < 50 years, < 30 mm/h for women 50–85 years, and < 42 mm/h for women over 85 years. Correspondingly, ESR was considered normal as < 15 mm/h for men < 50 years, < 20 mm/h for men between 50 and 85 years, and < 30 mm/h for men over 85 years, according to the laboratory reference values.

Serology. Patients were considered seropositive if they had a positive titer for rheumatoid factor (RF) and/or antibodies for anti-citrullinated proteins (ACPA), measured any time over disease course. A level of ≥ 15 IU/ml was considered elevated for RF according to the laboratory reference values. A level of ≥ 7 kU/l was considered elevated for ACPA according to the laboratory reference values.

Disease activity. Joint counts included swollen joint count (SJC) and tender joint count (TJC), on 28, 46, and 66/68 joints, assessed by the doctor, as well as the doctor’s global assessment of disease activity (Dr.global) on a visual analog scale (VAS) of 0–100 mm.

DAS28-ESR (DAS28), ranging from 0 to 9.4, was used to measure disease activity, with < 2.6 as the threshold value for remission.

cDAPSA was used to measure disease activity, in addition to DAS28. It includes patient global assessment (PGA) and pain, both on a scale of 0 to 10 cm as well as SJC on 66 joint count and TJC on 68 joint count, all summed together with a range of 0 to 154, with the cut-off point of ≤ 4 for remission, ≤ 13 for low disease activity, ≤ 27 for moderate disease activity, and > 27 for high disease activity [5].

DAPSA was also used to measure disease activity. It includes the same variables as cDAPSA and the CRP value.

Body surface area (BSA) measures the proportion of skin affected by psoriasis (0–100%) and was used as part of patient self-report. A value of < 3% equals mild, 3–10% moderate, and > 10% severe psoriasis [14]. A value of ≤ 3% is also used to describe remission in disease activity measurement tools for PsA [15].

Definitions of remission. In addition to the DAS28-definition of < 2.6 and cDAPSA-definition of ≤ 4, we also compared remission rates according to other definitions between PsA and RA. The following were included:

- Dr.global remission with a VAS score of 3/100 or less [10]
- Clinical remission on 28 joints (Clin28): no swollen/tender joints on SJC28/TJC28, and a normal age-specific level of ESR [10]
- Clinical remission on 46 joints (Clin46), no swollen/tender joints on SJC46/TJC46, and a normal age-specific ESR-level [10]
- Clinical Disease Activity Index (CDAI)—remission of ≤ 2.8, according to the formula PGA + Dr.global (both on a scale of 0–10) + SJC28 + TJC28 [16]
- Boolean definition for remission according to the ACR/EULAR remission criteria of ≤ 1 for SJC28, ≤ 1 for TJC28, a level of ≤ 10 mg/l for CRP, and a PGA of ≤ 1 on a scale of 1–10 [17]

Medication data were obtained from the national Drug Purchase Register for each patient. Medications were analyzed as “ever used” in case a patient purchased the medication any time since 1st January 2000 until the index date and “current use” if purchases happened 6 months prior to each individual’s index date.

Medications of interest included conventional synthetic DMARDs (csDMARDs) such as methotrexate (MTX), sulfasalazine (SSZ), hydroxychloroquine (HCQ), and leflunomide (LEF); prednisolone (GC); bDMARDs as one group; targeted synthetic DMARDs (tsDMARDs) such as Janus kinase inhibitors (JAK inhibitors) as one group; and apremilast, a phosphodiesterase-4 (PDE4) inhibitor.

### Specific aims

Our aim #1 was to study the overall current disease status of patients with PsA and RA in Finland using DAS28 and cDAPSA. Our aim #2 was to compare the mean disease activity level in patients with PsA and RA, in different sex and age groups, using both DAS28 and cDAPSA. Our aim #3 was to study whether there are regional differences in these disease activity measures in Finland. Our aim #4 was to compare the remission rates in PsA and RA using different definitions of remission. Finally, the aim #5 was to describe medications of patients with PsA and RA, to provide an overall picture of the medical care of the patients.

### Statistical methods

A value of *p* = 0.05 was set as a threshold for statistical significance. Categorical variables were described using frequency counts and percentages. Continuous variables were described using means and standard deviations or medians and interquartile ranges (IQR) depending on the way the variable is distributed. Chi-square test was used in the comparison of the use of DMARDs between patients with PsA and RA. Analysis of variance (ANOVA) was used to compare continuous variables.

Regression models were applied to compare measures of clinical status and remission rates between the groups, as crude analyses and with adjusting for age and sex. Continuous variables with skewed distributions (such as SJC46 and CRP) were dichotomized at the overall/combined median value and then compared between groups using logistic regression models.

Analyses were conducted using the R Statistical language (version 4.2.1; R Core Team, 2022) on Ubuntu 20.04.5 LTS.

### Ethical issues

This study was conducted as a register-based study using data from the Finnish Rheumatology Quality Register. It is kept by the THL, which granted approval for the study and the permission to use patient data for secondary purposes, being scientific research in this case. The data used in this study was pseudonymized. Patient consent was not required with this study setting.

## Results

### Demographics

A total of 3598 patients with PsA (51% female subjects) and 13,913 (72% female subjects, 74% ACPA-positive) patients with RA were identified from the national quality register for inflammatory arthritides between 1st January 2020 and 30th September 2021 (Table 1). Patients with PsA were younger: the mean (SD) age for patients with PsA was 54 (14) and 62 (14) for patients with RA (*p* < 0.001). The median (IQR) disease duration was 7 (2, 15) years for patients with PsA and 9 (3, 20) years for patients with RA (*p* < 0.001). The median (IQR) diagnostic delay was 11 (4, 36) months and 5 (3, 12) months, respectively (*p* < 0.001). A total of 16% of patients with PsA were current smokers and 15% of patients with RA (*p* = 0.169). The corresponding proportions for previous smokers were 39% and 34%, respectively (*p* < 0.001). A total of 2693 (75%) of patients with PsA were in working age (< 65 years old) and 6997 (50%) of patients with RA. Among patients with PsA who were in working age, a total of 69% were currently employed, 21% disabled, 7% unemployed, and 3% were not in work force. The corresponding numbers for patients with RA were 66%, 25%, 6%, and 4% (Table 1).

**Table 1:** Demographic and clinical variables of patients with PsA and RA

Variable	Available data for patients with PsA *n*, %	PsA	Available data for patients with RA *n*, %	RA	*p* value
Demographic variables					
* n*		3598		13,913	
Female subjects *n*, %	3598 (100%)	1843 (51%)	13,913 (100%)	10,038 (72%)	< 0.001
Mean age (SD)	3598 (100%)	54 (14)	13,913 (100%)	62 (14)	< 0.001
Median diagnostic delay in months (IQR)	950 (26%)	11 (4, 36)	4923 (35%)	5 (3, 12)	< 0.001
Median disease duration in years (IQR)	2457 (68%)	7 (2, 15)	10,777 (77%)	9 (3, 20)	< 0.001
Smoking status *n*, %	3267 (91%)		12,443 (89%)		
Current smokers		515 (16%)		1839 (15%)	0.169
Previous smokers		1278 (39%)		4286 (34%)	< 0.001
Proportion of patients that are < 65 years old *n*, %	3598 (100%)	2693 (75%)	13,913 (100%)	6997 (50%)	
Employment status in patients under 65 years old, *n*, %	1947 (72%)		5003 (72%)		
Employed		1351 (69%)		3290 (66%)	
Disabled		409 (21%)		1238 (25%)	
Unemployed		129 (7%)		291 (6%)	
Not in work force		58 (3%)		184 (4%)	
Clinical variables*					
ACPA-positive *n*, %	1834 (51%)	90 (5%)	10,284 (74%)	7648 (74%)	< 0.001
Median (IQR) CRP	2865 (80%)	2 (1, 5)	11,281 (81%)	3 (1, 6)	0.025
Median (IQR) ESR	2647 (74%)	7 (3, 14)	10,770 (77%)	8 (5, 18)	0.821
Median (IQR) SJC 46	2768 (77%)	0 (0, 1)	10,807 (78%)	0 (0, 1)	< 0.001
Median (IQR) TJC 46	2768 (77%)	0 (0, 2)	10,807 (78%)	0 (0, 2)	0.002
Proportions of patients with an SJC66-value of 0	2768 (77%)	1884 (68%)	10,807 (78%)	6674 (62%)	< 0.001
Proportion of patients with a TJC68-value of 0	2768 (77%)	1405 (51%)	10,807 (78%)	5554 (51%)	0.002
Median (IQR) Dr.global	2585 (72%)	8 (0, 18)	9614 (69%)	8 (0, 19)	0.003
Median (IQR) DAS28	2569 (71%)	1.9 (1.4, 2.6)	10,060 (72%)	2.0 (1.6, 2.7)	0.940
Mean (SD) DAS28	2569 (71%)	2.2 (0.8)	10,060 (72%)	2.3 (0.9)	< 0.001
Median (IQR) cDAPSA	2530 (70%)	7.7 (3.1, 14)	9279 (67%)	7.7 (3.3, 14)	< 0.001
Mean (SD) cDAPSA	2530 (70%)	9.5 (8.4)	9279 (72%)	9.7 (8.8)	0.234
Median (IQR) DAPSA	9279 (67%)	8.2 (3.7–14.0)	2396 (67%)	8.1 (3.5, 14.0)	< 0.001
BSA, %	1253 (35%)				
0		441 (35.2%)			
1–2		568 (45.3%)			
3–10		202 (16%)			
> 10		42 (3.4%)			

*Comparisons adjusted for age and sex for clinical variables

### Measures for disease activity in PsA and RA

Median (IQR) SJC46 was 0 (0, 1) for patients with PsA and 0 (0, 1) for patients with RA (*p* < 0.001). For TJC46, the corresponding numbers were 0 (0, 2) and 0 (0, 2) (*p* < 0.001). The proportion of patients with no swollen joints on SJC66 was 68% for patients with PsA and 62% for patients with RA (*p* < 0.001). The proportion of patients with no tender joints on TJC68 was 51% for PsA and 51% for RA (*p* = 0.002). The median (IQR) CRP was 2 (1, 5) for patients with PsA and 3 (1, 6) for patients with RA (*p* = 0.032). The median (IQR) ESR was 7 (3, 14) and 8 (5, 18), respectively (*p* = 0.79). The median (IQR) Dr.global was 8 (0, 18) for PsA and 8 (0, 19) for RA (*p* = 0.003). *p* values were adjusted for age and sex (Table 1).

Among PsA patients, 81% reported mild skin activity (BSA < 3), including 35.2% with no activity. Only 3.4% had BAS > 10 (Table 1).

### Comparison of disease activity on cDAPSA and DAS28 in PsA and RA, by age and sex

**Fig. 1:**
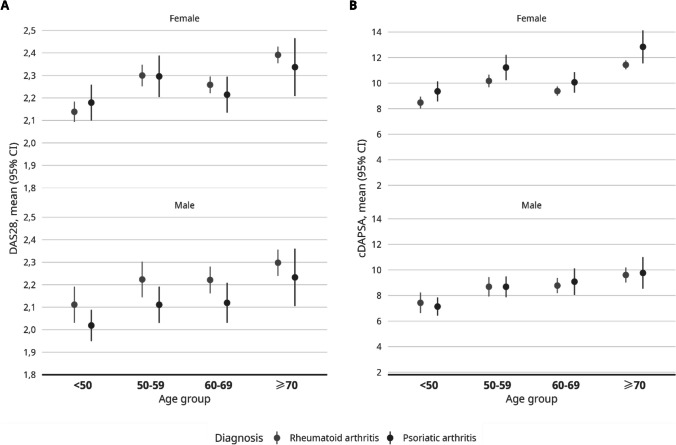
Mean (95% CI) DAS28 (**A**) and cDAPSA (**B**) by sex and by age in patients with PsA and RA

Mean disease activity on DAS28 was ≤ 2.4 in all age and sex groups, in both PsA and RA (Table 2, Fig. 1A). DAS28 was higher in RA versus PsA in men in all age groups as well as in women ≥ 60 years old. However, in women who were < 50 years old, the mean DAS28 was higher in patients with PsA. The difference was not statistically significant in any of the groups. The mean DAS28 was higher in older versus younger age groups in both women and men and in both diseases.

**Table 2:** Disease activity of patients with PsA and RA by sex and by age

Disease activity in different age and sex groups			
Mean (95% CI) DAS28 by age for male subjects			
< 50 years old	2.0 (1.9, 2.1)	2.1 (2.0, 2.2)	0.087
50–59 years old	2.1 (2.0, 2.2)	2.2 (2.1, 2.3)	0.051
60–69 years old	2.1 (2.0, 2.2)	2.2 (2.2, 2.3)	0.062
≥ 70 years old	2.2 (2.1, 2.4)	2.3 (2.2, 2.4)	0.361
Mean (95%CI) DAS28 by age for female subjects			
< 50 years old	2.2 (2.1, 2.3)	2.1 (2.1, 2.2)	0.382
50–59 years old	2.3 (2.2, 2.4)	2.3 (2.3, 2.3)	0.940
60–69 years old	2.2 (2.1, 2.3)	2.3 (2.2, 2.3)	0.329
≥ 70 years old	2.3 (2.2, 2.5)	2.4 (2.4, 2.4)	0.423
Mean (95% CI) cDAPSA by age for male subjects			
< 50 years old	7.1 (6.4, 7.8)	7.4 (6.6, 8.2)	0.593
50–59 years old	8.7 (7.9, 9.5)	8.7 (7.9, 9.4)	0.996
60–69 years old	9.1 (8.0, 10)	8.8 (8.2, 9.4)	0.614
≥ 70 years old	9.8 (8.5, 11)	9.6 (9.0, 10)	0.817
Mean (95% CI) cDAPSA by age for female subjects			
< 50 years old	9.4 (8.6, 10)	8.5 (8.0, 8.9)	0.057
50–59 years old	11.2 (10, 12)	10.2 (9.7, 11)	0.062
60–69 years old	10.1 (9.3, 11)	9.4 (9.0, 9.7)	0.122
≥ 70 years old	12.8 (12, 14)	11.4 (11, 12)	0.038

The mean cDAPSA was below 13 in all age and sex groups, in both PsA and RA (Table 2, Fig. 1B). No group had a mean value of < 4. For male subjects, the mean values were similar in PsA and RA in all age groups. In female subjects, the mean cDAPSA values were higher for PsA in all age groups, with a statistically significant difference in patients ≥ 70 years old (*p* = 0.038). The mean cDAPSA was higher in older versus younger age groups in both women and men and in both diseases.

### Disease activity on DAS28 in PsA and RA, by health care region

**Fig. 2:**
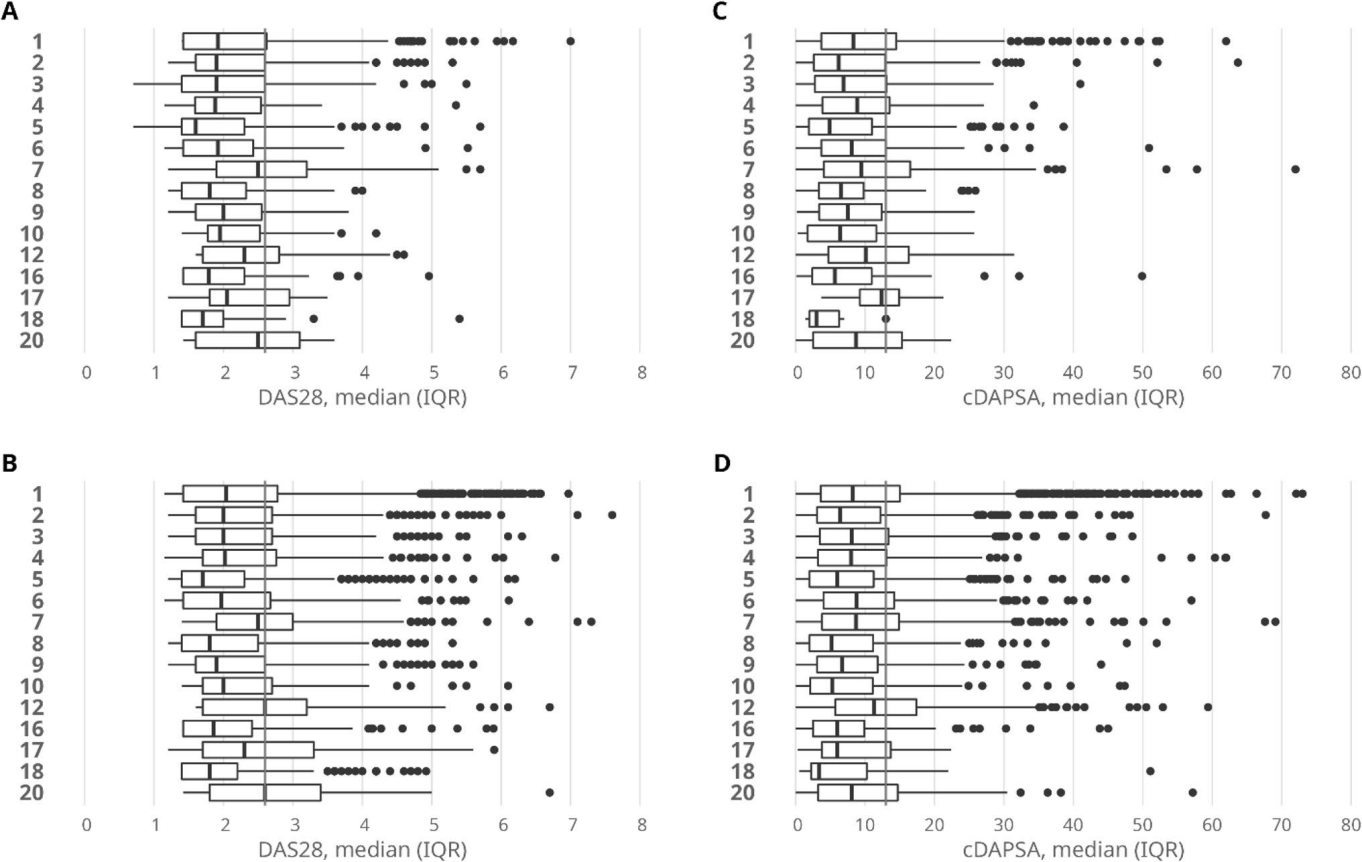
The median (IQR) DAS28 in PsA (**A**) and RA (**B**), and median (IQR) cDAPSA in PsA (**C**) and RA (**D**) by health care region in Finland. The reference line is at 2.6 for DAS28 and 13 for cDAPSA

In all health care regions and in both diseases, the median DAS28 was ≤ 2.6 (Fig. 2A, B).

In patients with PsA, the median (IQR) DAS28 was 1.9 (1.4, 2.6) and 73% had a score of < 2.6. Between regions, the median scores ranged from 1.6 (1.4, 2.3) to 2.5 (1.9, 3.2) (Fig. 2A) and the proportion of patients with a DAS28 score of < 2.6 ranged from 52% to 90% (*p* < 0.001, adjusted for age and sex).

In patients with RA, the median (IQR) DAS28 was 2.0 (1.6, 2.7) and 69% had a DAS28 score of < 2.6. Between regions, the median scores ranged from 1.7 (1.4, 2.3) to 2.6 (1.8, 3.4) (Fig. 2B) and the proportion of patients with a DAS28 score of < 2.6 ranged from 48% to 85% (*p* < 0.001, adjusted for age and sex).

Unadjusted median DAS28 values in PsA and RA in health care regions are illustrated in Fig. 2A, B.

### Disease activity on cDAPSA in PsA and RA, by health care region

In all health care regions and for both diseases, the median cDAPSA was < 13 (Fig. 2C, D).

In patients with PsA, the median (IQR) cDAPSA was 7.7 (3.1, 14.0) and it ranged from 4.9 (1.9, 11.0) to 12.0 (9.2, 15.0) between regions (Fig. 2C). A total of 32% of all patients had a cDAPSA score of ≤ 4, and 42% were in a group of low disease activity, 23% in moderate disease activity, and 4% in high disease activity. The proportion of patients with remission or low disease activity (cDAPSA ≤ 13) varied from 63 to 93% between regions (*p* < 0.001, adjusted for age and sex).

In patients with RA, the median (IQR) cDAPSA was 7.7 (3.3–14.0) and it ranged from 5.2 (2.0, 11.0) to 11.0 (5.8, 17.0) between regions (Fig. 2D). A total of 30% had a cDAPSA score of ≤ 4, 43% were in a group of low disease activity, 23% in moderate disease activity, and 4% in high disease activity. The proportion of patients with remission or low disease activity (cDAPSA ≤ 13) varied from 57% to 86% between regions (*p* < 0.001, adjusted for age and sex).

Unadjusted median cDAPSA values in PsA and RA in health care regions are illustrated in Fig. 2C, D.

### Remission rates by different definitions in PsA and RA

**Fig. 3:**
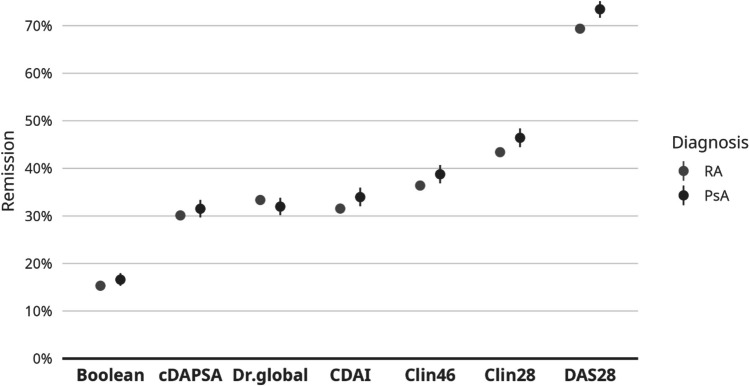
Remission rates in PsA and RA, according to different definitions of remission

Remission rate according to the ACR/EULAR Boolean definition was 17% for patients with PsA and 15% for patients with RA (*p* = 0.001). Correspondingly, the proportions were 32% and 30% for cDAPSA (*p* < 0.001), 32% and 33% for Dr.global (*p* < 0.001), 34% and 32% for CDAI (*p* = 0.004), 39% and 36% for Clin46 (*p* = 0.761), 46% and 43% for Clin28 (*p* = 0.901), and 73% and 69% for DAS28 (*p* = 0.171) (Table 3, Fig. 3) Comparisons were adjusted for age and sex.

**Table 3:** Remission rates by different definitions in patients with PsA and RA

Variables	Available data for patients with PsA *n*, %	PsA, *n* = 3598	Available data for patients with RA *n*, %	RA, *n* = 13.913	*p* value*
Remission rates by different definitions					
Boolean	3170 (88%)	17%	12,317 (89%)	15%	< 0.001
cDAPSA	2530 (70%)	32%	9279 (67%)	30%	< 0.001
Dr.global	2585 (72%)	32%	9614 (70%)	33%	< 0.001
CDAI	2309 (64%)	34%	8543 (61%)	32%	0.004
Clin46	2583 (72%)	39%	10,396 (75%)	36%	0.761
Clin28	2546 (71%)	46%	10,287 (74%)	43%	0.901
DAS28	2569 (71%)	73%	10,060 (72%)	69%	0.171

*Comparisons adjusted for age and sex

### Previous and current use of DMARDs and GCs

MTX was the most widely used DMARD for both PsA and RA, with 89% and 91% of patients (*p* < 0.001) having used it previously and 49% and 57% of patients currently using it (*p* < 0.001). The corresponding proportions for subcutaneous MTX users were 53% and 48% (*p* < 0.001) and 24% and 23% (*p* = 0.380) (Table 4).

**Table 4:** Self-administered DMARDs in patients with PsA and RA; “ever used”: medication purchased between 1st January 2000 and the index date; “current use”: medication purchased during a 6-month period prior to the index date

	PsA *n* = 3598 % of patients		RA *n* = 13,913 % of patients		*p* ever use	*p* current use
	Ever used	Current use	Ever used	Current use		
Any DMARD (no Pred)	93	83	96	86	< 0.001	< 0.001
Methotrexate, overall	89	49	91	57	0.007	< 0.001
p.o	83	27	85	36	< 0.001	< 0.001
s.c	53	24	48	23	< 0.001	0.380
Sulfasalazine	51	13	63	27		
Hydroxychloroquine	15	1.7	80	45		
Leflunomide	26	6.1	31	7.2		
Prednisolone	69	16	90	38		
bDMARDs	44	37	32	21	< 0.001	< 0.001
JAK inhibitors	3.3	1.9	6.6	4.6	< 0.001	< 0.001
PDE4 inhibitor	7.6	3.4	-	-		

Secondary csDMARDs such as SSZ, LEF, and GCs were previously used by 51%, 15%, and 69% of patients with PsA, with 13%, 6.1%, and 16% as the corresponding proportions for current users.

bDMARDs were “ever used” by 44% of patients with PsA and 32% of patients with RA (*p* < 0.001). They were currently used by 37% of patients with PsA and 21% of patients with RA (*p* < 0.001). Among tsDMARDs, the proportions were 3.3% and 6.6% for current use (*p* < 0.001) and 1.9% and 4.6% for “ever” use (*p* < 0.001). PDE4-inhibitors were “ever” used by 7.6% and currently used by 3.4% of patients with PsA (Table 4).

## Discussion

### Disease activity in PsA and RA

Our main observation was that the current disease activity was low and similar in PsA and RA across Finland, indicated by using various measures. Majority of patients with RA were in remission by DAS28 scores (69%) and most patients with PsA were in remission or had low disease activity by cDAPSA scores (74%), suggesting that current treatment goals have been achieved in most patients in Finland in the current decade. The disease activity was also almost identical between PsA and RA in all age and sex groups, measured by DAS28 and cDAPSA, along with high proportion of patients with no symptomatic joints and similar joint status between patients with PsA and RA. For both measures, there was only some variation between regions (Fig. 2).

To our knowledge, only three earlier studies compared disease activity between PsA and RA and all of them showed similar activity between the diseases. Our results were similar with a recent Danish study [18] with a mean DAS28 of 2.2 in PsA (1151 patients) and 2.1 in RA (4990 patients) versus a mean DAS28 of 2.2 in PsA and 2.3 in RA in our study (Table 1). A study from Norway in 2015 [19] presented a median DAS28 of 2.9 for patients with PsA and 2.6 for patients with RA, whereas our corresponding median values were 1.9 and 2.0 (Table 1). In a study from the USA conducted in 2019, the mean DAS28 was 3.5 in PsA and 3.7 in RA [20].

In contrast to a recent cross-sectional Danish study of 197 patients with PsA [21], the overall median DAPSA score of 15 was noticeably higher compared to our study with a DAPSA of 8.1.

### Remission rates in PsA and RA

The remission rates ranged from 17% to 73% in patients with PsA and from 15% to 69% in patients with RA, depending on the definition that was used. The remission rate was quite similar in both diseases among each definition (Table 3, Fig. 3) and the maximal difference was only 4% between PsA and RA, namely, in DAS28 remission. In three of the definitions (cDAPSA, CDAI, and Boolean remission), the remission rate was statistically significantly higher in PsA versus RA, but it was opposite in Dr.global. DAS28 had the highest remission rates (73% and 69%) and Boolean definition the lowest (17% and 15%). The other remission rates ranged around 30–46%, which were in line with Dr.global ≤ 3 (32% and 33%), indicating that probably one third of patients are in remission according to a physicians’ opinion in general, and cDAPSA, CDAI, and Clin46 reflect the physicians’ opinion in terms of remission.

The lowest remission rates were seen for the Boolean definition. The PGA component has been shown to increase due to non-arthritis related factors such as psychosocial factors or fatigue [22]. A requirement of PGA (≤ 1/10 cm) has been argued to be too stringent, since it has been shown to be in discordance with other clinical components of the definition for Boolean remission, being the only limiting factor for remission [23–25]. Furthermore, in elderly non-RA population, the overall mean PGA is about 20/100 mm [26]. Other definitions for remission did not include PGA, excluding CDAI and DAS28, in which the influence of PGA to the total score is minor. The overall median (IQR) values for other clinical variables such as SJC, TJC, and laboratory markers were mostly normal and there were no noticeable differences between patients with PsA and RA. Most patients had no swollen joints and about half of the patients had no tender joints in the SJC66/TJC68.

Remissions are rarely reported in PsA. An exception is a Norwegian PsA study from 2017 [27], with a proportion of cDAPSA remission or low disease activity of 70%, compared to 75% in our patients.

On contrary to PsA, remission rates are often reported in RA. In a cross-sectional study of several countries in the 2000s, the DAS28 remission rate was as low as 20% [10]. However, more recent studies have shown that rates of > 50% are not unusual in cross sectional settings [28] and in patients with early disease, even > 70% [29]. Remission rates according to other definitions seemed to be significantly higher in this study compared to the previous study on RA patients, with a difference of approximately 7–30% depending on the variable [10].

### Medications

The anchor drug for the treatment of both diseases was MTX and secondary csDMARDs were used according to the national treatment regimen. The overall use of bDMARDs was lower for our patients with RA compared to a recent cross-sectional Norwegian study (21% versus 27%) [30]. On the contrary, bDMARDs were used significantly more in our patients with PsA (37%) than in Norwegian PsA patients (26%) [27, 30]. Though, in Finland, due to a shared care between rheumatology clinics and primary health care, patients who stay in remission with csDMARDs are being followed by primary health care. On the contrary, patients on bDMARDs are more likely to be under monitoring by rheumatology clinics, which may have caused a concentration of patients using bDMARDs in our study population.

### Strengths and limitations

A strength of this study was its comprehensive coverage of almost all hospital districts in Finland. A large and heterogenous pool of patients from all geographical sites also enables an accurate depiction of the current disease activity and treatments.

Limitations due to a cross sectional observational setting of a register-based study. Typical to observational studies, data were not complete for all variables, although available for the majority of variables in > 70% of the patients. Also, 5 hospital districts had to be excluded from the analyses of regional differences due to almost non-existent patient coverage. Furthermore, due to an observational setting, no firm conclusions for causal explanations can be drawn.

Limitations concerning measures. As for the different definitions of remission, most of them were not validated tools for describing disease activity in both PsA and RA. Furthermore, these definitions do not take the extra-articular aspects of PsA into account, which might contribute to the overall disease burden. However, these same clinical aspects are not found in RA and therefore other, more feasible definitions of PsA remission [15] cannot be used in comparative studies between PsA and RA. Nevertheless, according to the BAS, 81% of PsA patients had none or mild skin activity only.

Other limitations. The number of patients using bDMARDs would have been somewhat higher if those receiving treatment via infusions were included. Concerning diagnoses, they were based on the treating rheumatologists’ judgment on clinical basis, with the help of the classification criteria at the time of the diagnosis. Also, a quarter of patients with RA were seronegative, which has been shown to have a different clinical picture in the long-term follow-up compared to its seropositive counterpart [31]. Furthermore, the subtype of PsA was not available in the database.

## Conclusions

This study provides comprehensive cross-sectional data from the nationwide quality register in Finland, showing that most patients with PsA and RA are doing well in terms of disease activity, by several measures. Furthermore, no major differences were seen in terms of common clinical variables such as SJC 66, TJC 68, ESR, CRP, Dr.global, and disease activity between these diseases, as well as across regions. Our results are in line with other Nordic countries concerning low disease activity levels. Although these results are promising, a proportion of patients with PsA and RA still have active disease, indicating that there is still room for improvement.
